# Co-Design of Smartphone- and Smartwatch-Based Occupational Health Visualisations in Office Environments

**DOI:** 10.3390/s26072278

**Published:** 2026-04-07

**Authors:** Phillip Probst, Sara Santos, Gonçalo Barros, Mariana Morais, Sofia Garcia, Philipp Koch, Jorge Barroso Dias, Ana Leal, Rute Periquito, Sofia André, Tiago Matoso, Cristina Pinho, Ricardo Vigário, Hugo Gamboa

**Affiliations:** 1 LIBPhys (Laboratory for Instrumentation, Biomedical Engineering and Radiation Physics), Department of Physics, NOVA School of Science and Technology, NOVA University Lisbon, Largo da Torre, 2829-516 Caparica, Portugal; slm.santos@fct.unl.pt (S.S.); mp.morais@campus.fct.unl.pt (M.M.); sp.garcia@campus.fct.unl.pt (S.G.); r.vigario@fct.unl.pt (R.V.); hgamboa@fct.unl.pt (H.G.); 2 Coimbra Institute for Biomedical Imaging and Translational Research (CIBIT), Instituto de Ciências Nucleares Aplicadas à Saúde (ICNAS), University of Coimbra, R. Santa Comba, Celas, 3000-548 Coimbra, Portugal; uc50034@uc.pt; 3 German Research Center for Artificial Intelligence (DFKI), 23562 Lübeck, Germany; philipp.koch@dfki.de; 4 DSHS (Departamento de Saúde, Higiene e Segurança), Câmara Municipal de Lisboa, Avenida Ressano Garcia 2, 1070-228 Lisbon, Portugal; jorge.barroso.dias@cm-lisboa.pt (J.B.D.); rute.periquito@cm-lisboa.pt (R.P.); sofia.andre@cm-lisboa.pt (S.A.); tiago.matoso@cm-lisboa.pt (T.M.); cristina.pinho@cm-lisboa.pt (C.P.)

**Keywords:** co-design, data visualisation, office environments, occupational health, biomedical engineering

## Abstract

Office workers are exposed to a range of occupational health risks, including prolonged sedentary behaviour, postural load, elevated heart rate, and noise, yet objective and continuous monitoring of these risk factors in workplace settings remains uncommon. This study aimed to co-design occupational health visualisations based on smartphone and smartwatch data, through a multi-stakeholder group of office workers and occupational health professionals. A generative co-design framework was applied, comprising a pre-design phase with a field study and questionnaire, a structured multi-stakeholder workshop, and a follow-up evaluation session. Thematic analysis of the workshop transcript yielded 17 occupational health themes, which were subsequently assessed for technical feasibility relative to the available sensing platform. Of the 27 discrete visualisation elements proposed across both groups, the majority were classified as directly addressable using smartphone and smartwatch sensor data. Visualisations covering physical activity, heart rate, environmental noise exposure, and postural load were implemented in Python using real-world data collected from office workers. The follow-up session provided qualitative confirmation that the developed visualisations were interpretable and aligned with the stakeholder expectations. The generative co-design framework proved well-suited to the occupational health visualisation context, enabling structured translation of stakeholder requirements into technically feasible and interpretable visualisation outputs.

## 1. Introduction

People invest a major portion of their lives in paid employment, thereby subjecting themselves to motor, physiological, and mental adaptations that can significantly shape their long-term health status. Prolonged exposure to these factors can induce work-related disorders (WRDs). WRDs encompass a broad spectrum of conditions, ranging from musculoskeletal complaints to psychosocial conditions [1,2]. Among WRDs, work-related musculoskeletal disorders (WRMSDs) are the most prevalent category, representing the leading cause of absenteeism, productivity losses, and associated societal costs in Europe [2,3]. The incidence of WRMSDs is not confined to physically demanding occupations, as they are increasingly prevalent in office environments. In this context, prolonged static postures, repetitive upper-limb movements, and sustained sedentary behaviour represent well-established risk factors [1,4,5]. According to Eurostat, approximately 39% of employed individuals in Europe spend the majority of their working time seated [6]. The associated risks extend well beyond the musculoskeletal domain, leading also to cardiovascular and metabolic consequences [5,7]. Beyond musculoskeletal risk, office workers are further exposed to psychosocial stressors, elevated ambient noise levels, and inadequate ergonomic conditions, all of which can adversely affect health and well-being [8,9].

Occupational health assessments, which aim to identify and mitigate potential risk factors before they manifest as disease, are becoming more prevalent. In practice, however, these assessments are typically conducted by trained experts within a limited time frame, relying on observational or self-reported measures to evaluate workstations and working conditions [4]. Continuous information about motor and physiological adaptations occurring during the working day is rarely collected, leaving occupational health professionals without objective longitudinal data upon which to base their recommendations [10,11].

The widespread adoption of consumer-grade wearable technology offers a promising means of addressing this data gap. Smartphones and smartwatches are now ubiquitous in daily life and are equipped with an extensive array of embedded sensors. These include accelerometers, gyroscopes, and optical heart rate monitors, enabling the continuous, unobtrusive acquisition of physiologically and behaviourally relevant data [12,13]. Several studies have demonstrated the feasibility of using smartphone accelerometers to classify activity states, estimate postural load, and detect prolonged sedentary episodes in occupational contexts [14,15,16]. A growing body of work has further explored smartphone-based interventions to reduce sedentary behaviour in office workers, with results indicating modest but consistent reductions in sitting time [15,17,18]. The internal microphone of the smartphone has also been explored as an instrument for estimating ambient noise levels [19,20]. Studies have shown that calibrated smartphone microphones can achieve measurement accuracy with reasonable error margins to certified reference instruments [19,21,22]. Smartphone- and smartwatch-based applications for occupational and general health monitoring have also been reviewed more broadly, confirming the potential of these platforms to support health monitoring and risk identification in workplace settings [23,24]. Despite this potential, consumer-grade devices have not yet been leveraged to deliver multimodal occupational health visualisations that integrate data from multiple risk domains simultaneously.

Even where sensor data are available, their communicative value depends critically on how they are presented. Raw physiological and kinematic signals are difficult to interpret without specialist expertise. Health professionals and workers alike may lack the background required to extract meaningful, actionable insights from the data [25,26]. Data visualisations can serve as a common language between professionals and those that need to interpret these. Thus, translating complex sensor outputs into representations that can inform individual behavioural change, support clinical assessment, and facilitate evidence-based decision-making at the organisational level is necessary [27]. For occupational health applications specifically, effective visualisations must balance technical accuracy with interpretability across diverse stakeholders, such as workers, occupational health specialists, ergonomists, and managers. Consequently, these diverse groups of stakeholders may bring different levels of data literacy and distinct informational needs.

Co-design has emerged as a methodological approach for ensuring that digital health products are grounded in the needs, expectations, and contextual realities of those who will use them [28,29,30]. Generally speaking, co-design can be defined as the structured involvement of end users and domain experts in the iterative design of tools or interventions. Within the occupational health domain, participatory approaches have been shown to raise awareness of musculoskeletal risk factors, promote stakeholder ownership of designed solutions, and improve the probability of adoption [31]. Applying co-design specifically to the development of occupational health data visualisations ensures that the resulting representations are both technically feasible and practically meaningful.

The present work was conducted within the framework of the Prevention of Occupational Disorders in Public Administrations based on Artificial Intelligence PLUS (PrevOccupAI+) project, which aims to characterise daily working activities, working conditions, and potential risk factors on an individual and organisational level within the Public Administration. Data collection within PrevOccupAI+ is performed using a consumer-grade Android smartphone (Xiaomi Redmi Note 9, Beijing, China) placed on the chest and a smartwatch (OPPO watch 41 mm, Dongguan City, China) worn on the wrist. These devices provide access to inertial, acoustic, and cardiovascular sensor streams without the need for dedicated research-grade instrumentation [32,33]. The co-design study described in the present work was carried out in collaboration with the Câmara Municipal de Lisboa (CML), a Portuguese local government body, whose occupational health specialists, ergonomists, psychologists, and customer service managers participated in the co-design process.

The present paper makes the following contributions:

- The application of the generative co-design framework for healthcare innovation proposed by Bird et al. [28] to the development of multimodal occupational health data visualisations in an office environment. To the best of our knowledge, this work is among the first structured co-design studies of this kind in the context of office work.
- A systematic feasibility assessment of the participant-generated visualisation concepts against the sensor capabilities of the PrevOccupAI+ consumer-grade sensing platform, classifying each proposed concept as directly addressable, partially addressable, or out of scope.
- The implementation of an initial set of eight smartphone- and smartwatch-based occupational health visualisations in Python, covering the domains of physical activity and sedentary behaviour, heart rate, environmental noise exposure, and postural load.
- A qualitative assessment of the developed visualisations through a follow-up session with the co-design workshop participants in terms of interpretability and clarity of the visual design.

The remainder of this paper is organised as follows. Section 2 reviews related work on co-design in digital health and occupational ergonomics, and on the visualisation of occupational health data. Section 3 describes the materials and methods, encompassing the co-design framework, the pre-design phase, the co-design workshop, the post-design analysis, and the development of the visualisations. Section 4 presents the results. Section 5 discusses the findings, limitations, and directions for future work. Section 6 concludes the paper.

## 2. Related Work

### 2.1. Co-Design in Digital Health and Occupational Ergonomics

The involvement of end users and domain experts in the iterative design of digital health tools has gained substantial recognition as a means of improving the relevance, usability, and adoption of health interventions [29,34]. Participatory approaches, which range from consultative user studies to fully generative co-design workshops, share the objective of grounding system design in the lived experience of those who will ultimately use the resulting tools [28,29,30]. The principal benefit of structured co-design is that it surfaces implicit needs and contextually relevant design requirements that are unlikely to emerge from expert-driven development alone [29,34]. A systematic review by Duffy et al. [34] identified collaboration between heterogeneous stakeholder groups, in situ contextualisation of design activities, and iterative testing as the defining characteristics of successful co-design in digital health.

Within the occupational domain, Gyi et al. [31] demonstrated that co-developing ergonomic interventions with business drivers and their managers led to raised awareness of musculoskeletal symptoms affecting the workforce, successfully inducing change at the management level. More recently, Branco et al. [35] conducted a two-phase study combining a focus group with six co-design workshops to develop customisable clinical dashboards for multidisciplinary care teams managing Parkinson’s disease. The study engaged a clinically diverse group of fifteen healthcare professionals, namely physiotherapists, speech therapists, occupational therapists, and nurses, alongside HCI researchers. The co-design process revealed distinct visualisation priorities across clinical disciplines while identifying shared interests in role-adaptable granularity, dynamic dashboard customisation, and structured information sharing between team members. The work underscores that co-designing visualisations with multidisciplinary stakeholders from the outset of the design process is essential for ensuring that data representations align with the specific workflows and informational needs of different professional roles.

### 2.2. Data Visualisation of Occupational Health Risk

Efforts to visualise occupational health data for office workers have emerged across several distinct risk domains. However, these have largely remained isolated to individual health dimensions rather than providing an integrated multimodal view of occupational risk.

In the domain of workplace stress, Stepanovic et al. [27] developed and piloted a set of physiolytics-based visualisations for a Swiss public administration municipality. Physiological data was collected via wearable biosensors to generate aggregate-level representations of employee stress patterns. The study demonstrated that data visualisations can provide meaningful and evidence-based organisational insights regarding occupational stress that potentially can be translated into change management strategies. Similarly, ambient noise at the workplace, which is a recognised contributor to stress and cognitive impairment in open-plan offices [9,36,37], has been explored as a target for situated visualisation [38]. This work conducted design workshops with office workers to elicit requirements for ambient noise displays and derived first-generation visualisation mockups for personal and shared desk-level displays. While this work illustrates the value of participatory requirement elicitation, it relied on dedicated custom microphone and display hardware.

Postural risk in office environments has received considerable attention in the ergonomics literature, with structured assessment tools such as the Rapid Office Strain Assessment (ROSA) [39] providing observational frameworks for ergonomic risk quantification. More recent work has sought to automate posture assessment using wearable inertial measurement units (IMUs). Martins et al. [40] presented ErgoReport, a holistic posture assessment framework that combines IMU-derived joint kinematics with deep learning-based posture classification to generate interpretable ergonomic risk reports via a graphical user interface. While ErgoReport represents a significant advance in automating and communicating ergonomic risk, its sensing configuration relies on dedicated research-grade IMU systems rather than consumer devices. Furthermore, its design was not informed by an end-user co-design process.

With regard to the usage of consumer-grade devices, such as smartphones and smartwatches, sedentary behaviour and physical inactivity in office workers have been the most extensively investigated topics. Multiple studies have demonstrated the feasibility of smartphone accelerometers for monitoring work activity patterns and delivering real-time prompts to interrupt prolonged sedentary behaviour [14,15,16,17]. For example, the Walk@Work mHealth intervention [18] used a smartphone application to monitor occupational sitting and standing, yielding moderate reductions in sedentary time during the intervention period. Just-in-time adaptive interventions (JITAIs) [41] have extended this approach by personalising prompt delivery based on real-time sedentary detection. A systematic review of digital workplace interventions targeting sedentary behaviour [15] confirmed that smartphone- and wearable-based tools can reduce sitting time, but highlighted inconsistency across studies in sensor configuration, outcome measurement, and the extent of end-user involvement in tool design. However, these interventions are predominantly single-domain in scope, mainly addressing physical activity in isolation from other occupational health dimensions.

The SWELL project [42] represents the most comprehensive attempt to date to build a multimodal, sensor-informed occupational health support system for knowledge workers. SWELL combined computer interaction logging, Kinect-based posture tracking, physiological wearables, and ecological momentary assessment to model stress and workload, as well as to develop personalised coaching interventions. The project demonstrated that multimodal sensing enables richer and more accurate health state modelling than any single modality in isolation. It explicitly called for approaches that address the full temporal complexity of occupational exposure. However, SWELL relied on a laboratory-based data collection environment, employed a mix of non-portable and research-grade sensors, and did not employ a co-design process to elicit end-user visualisation requirements.

The presented approaches demonstrate that occupational health monitoring in office environments has advanced considerably. Nevertheless, a clear gap persists: no existing work integrates a structured co-design methodology with the development of multimodal occupational health visualisations grounded in consumer-grade, widely accessible sensing hardware. Existing visualisation systems address isolated dimensions, such as noise, stress, posture, or sedentary behaviour. These typically depend on dedicated hardware, proprietary sensing platforms, or laboratory-grade equipment. mHealth interventions exploit smartphone sensors but are narrowly focused on physical activity and do not leverage the broader sensor array available. Furthermore, to the best of our knowledge, a structured co-design process for occupational health data visualisation, including workers and occupational health professionals, has not been reported yet within the domain of office work. Thus, the present study positions itself among the first to address this gap by applying the generative co-design framework [28] to identify multimodal occupational health visualisation requirements. Additionally, the identified requirements are used to develop visualisations using data acquired exclusively through the sensor modalities available on a consumer-grade Android smartphone and smartwatch.

## 3. Materials and Methods

### 3.1. Co-Design Framework

The generative co-design framework for healthcare innovation, proposed by Bird et al. [28], was selected to guide the study design. This framework was chosen for its structured yet flexible approach to end-user engagement in digital health contexts. It explicitly supports iterative requirement translation from participant-generated insights into implementable technical solutions. The framework organises the co-design process into seven steps distributed across three phases: (1) pre-design, (2) co-design, and (3) post-design.

The pre-design phase comprises two steps. First, a contextual inquiry was conducted to frame the occupational context and identify key challenges through observational fieldwork and an informational questionnaire. In the second step, the co-design phase was prepared through participant selection, facilitator assignment, and the allocation of preparatory materials.

The co-design phase was carried out as a structured three-hour workshop, comprising three steps. The workshop began by framing the identified issues through a presentation of the contextual inquiry findings, allowing participants to share their experiences and visions. This was followed by a generative design session in which participants were encouraged to brainstorm and develop low-fidelity visualisation prototypes representing solutions to the discussed occupational health challenges. In the final step, participants presented and discussed their concepts with the broader group. The workshop was audio-recorded to facilitate subsequent analysis.

The post-design phase encompassed the final two steps: data analysis and requirement translation. The audio recordings were transcribed, coded, and organised to identify key thematic categories. The resulting insights were subsequently translated into design requirements to guide the implementation of the proposed visualisations.

### 3.2. Pre-Design Phase

#### 3.2.1. Field Study

A field study was carried out in April 2025, prior to the distribution of the questionnaire, to obtain a detailed understanding of the contexts in which the public administration workers of CML performed their daily tasks. During the study, 11 offices spread across four locations were visited, encompassing both front-office and back-office environments. For each location, a predefined set of general features was systematically documented, including the number of workers, equipment in use, environmental factors such as lighting and noise, floor level, and the services provided. Photographs were taken at each location for post-analysis purposes.

Where workers were available and not attending to citizens, informal conversations were encouraged to gather subjective impressions of the workplace and working conditions. The availability of workers for conversation varied across offices, as several locations were actively engaged in citizen-facing services at the time of the visit. The primary objective of the field study was not to inform questionnaire design, but rather to observe the diversity of working contexts and to identify potential occupational pain points.

#### 3.2.2. Questionnaire

A questionnaire was developed in collaboration with CML and distributed via email to potential workshop participants in April 2025. Its primary purpose was to sensitise participants to occupational health topics prior to the workshop and to inform the design of the following workshop structure. Participants were selected by CML to ensure diversity in professional roles and years of experience within the organisation. The questionnaire was distributed to 14 individuals, of whom ten responded, corresponding to a response rate of 71 %. The questionnaire was implemented in LimeSurvey [43] to facilitate distribution and data collection.

The questionnaire was written in European Portuguese, and responses were collected and analysed in the same language. It was organised into five thematic sections: (1) general information, covering professional role, years of experience, and health data consultation habits; (2) current challenges and needs, addressing the perceived difficulty of interpreting health data and existing gaps; (3) data interpretation and visualisation preferences, exploring preferred visualisation types and update frequencies; (4) understanding occupational risks through data visualisation, examining awareness of workplace risks and the perceived utility of risk visualisations; and (5) collaboration between stakeholders and decision-making, investigating how health data is currently communicated across organisational roles. The questions consisted of multiple-choice, Likert scale, and open-answer formats.

The full translation of the questionnaire to English is provided in Table A1 in Appendix A.1. The corresponding results are summarised in Appendix A.2. Its findings are not interpreted as primary research results, but are presented for transparency and completeness.

### 3.3. Co-Design Workshop

#### 3.3.1. Location and Participants

The co-design workshop was held at the premises of CML in May 2025. Participants were recruited from among the questionnaire respondents by CML, based on three criteria: availability, role diversity, and years of experience within the organisation. An explicit effort was made to ensure that the selected participants represented a broad range of professional roles, so that the resulting design insights would reflect the perspectives of multiple stakeholder groups within the occupational health context and the organisation.

A total of 12 participants (two male, ten female) took part in the workshop. Table 1 presents each participant’s assigned identifier, professional role, gender, and years of experience within CML. The group included occupational health and safety specialists, an ergonomist, psychologists, a health professional, and customer service managers.

#### 3.3.2. Structure of the Workshop

The co-design workshop was structured into five parts: (1) presentation of co-design objectives and project overview, (2) framing of the identified issues through presentation of questionnaire insights, (3) brainstorming and idea summarisation, (4) co-design of visualisations, and (5) group presentations and plenary discussion of the co-designed visualisations. The workshop was facilitated by members of the research team, who were responsible for organising the overall structure and guiding the session. During the brainstorming and co-design activities, the facilitators adopted a non-directive role, remaining available exclusively to answer participant questions regarding the functionality and data capabilities of the utilised sensors. Before the workshop commenced, all participants were asked to sign an informed consent form explaining the necessity of audio recording the session. Participants were seated in a semicircle, with a Zoom H6 (Tokyo, Japan) stereo microphone audio recorder positioned approximately at its centre.

**Figure 1:**
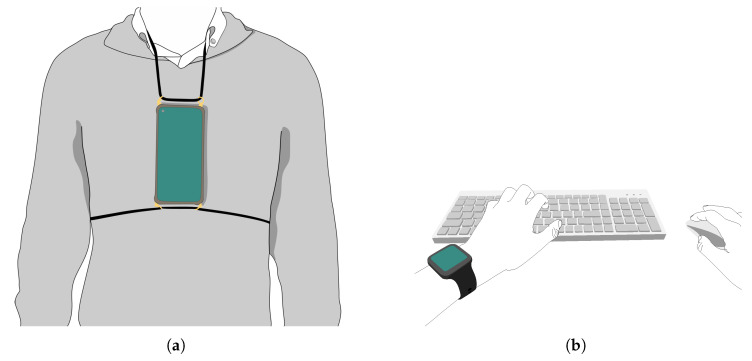
Device placement within the PrevOccupAI+ setup as presented to the co-design workshop participants. (**a**) Smartphone (Xiaomi Redmi Note 9, Beijing, China); (**b**) Smartwatch (OPPO watch 41 mm, Dongguan City, China).

The workshop opened with a 30-min presentation introducing the PrevOccupAI+ project, defining the objectives of the workshop, and explaining the relevance of data visualisation for communicating occupational risk factors at both individual and organisational levels. This was followed by a description of the utilised sensors, their placement on the worker as shown in Figure 1, and their respective data acquisition procedures. With regard to the acquisition procedure, it was explained that the phone acquires data throughout the working day, while the smartwatch is set to acquire at four instances of 20 min, due to battery limitations of the device. The presentation concluded with an overview of the types of information extractable from each device, as summarised in Table 2.

Following the presentation, the insights obtained from the questionnaire were shared in a 15 min plenary session. This was done to provide participants with a shared understanding of how technology and health data visualisations were currently used across different departments within CML, while also highlighting existing gaps and challenges.

For the brainstorming session, participants were divided into two groups of six. Each group was given 30 min to discuss and identify which occupational risk factors they considered most relevant to prioritise. To support a flexible ideation process, each group was provided with A2 white paper, A4 coloured paper, pencils, ballpoint pens, markers, post-it notes, scissors, and glue. Following the brainstorming session, a 30 min plenary discussion was held to consolidate the proposed risk factors across groups, from which four main thematic categories were identified to focus subsequent prototyping efforts.

For the co-design of data visualisations, participants were reorganised into two new mixed groups of six, ensuring that the brainstorming group composition was not replicated. Each group was allocated 45 min to develop visualisation prototypes for each of the four identified categories. Participants were explicitly encouraged to be creative and not to constrain their ideas based on perceived technical limitations. The same materials as used in the brainstorming session were provided. Upon completion, 20 min was allocated for group presentations and plenary discussion, with each group given ten minutes to present and discuss their developed visualisations.

The workshop concluded with a 10 min debrief that summarised the key outcomes of the session, after which participation certificates were presented to all participants.

### 3.4. Post-Design Phase

To ensure a robust and transparent qualitative analysis of the co-development sessions, a multi-stage process comprising transcription, verification, inductive coding, and thematic organisation was carried out. This process is described in the following sections.

#### 3.4.1. Transcription and Diarisation

The audio recording of the co-design workshop was split into sections of 15 to 20 min prior to transcription, retaining only those segments in which participant dialogue occurred. Segments consisting exclusively of facilitator-led presentations were excluded from the transcription pipeline. Transcription was performed using WhisperX [44], an open-source automatic speech recognition (ASR) system, configured for European Portuguese using a wav2vec2-based forced alignment model [45]. The standard model configuration was used. Although WhisperX supports automatic speaker diarisation, the diarisation output was found to be of insufficient quality for the present recording and was therefore discarded.

To validate and enhance the accuracy of the machine-generated transcripts and to perform speaker diarisation, two researchers conducted an independent proofreading and correction process. Each researcher independently (1) verified semantic coherence and corrected transcription errors, and (2) manually assigned speaker identifiers and precise timestamps to each utterance, ensuring transcript consistency and the preservation of the speakers’ authentic voice. The two independently corrected transcripts were subsequently merged into a single unified transcript using ChatGPT (GPT-4.1) [46]. This step was undertaken solely to accelerate the merging process. The resulting unified transcript was independently reviewed and approved by both researchers prior to any further analysis. Any discrepancies between the two independently corrected transcripts were resolved through discussion between the researchers until consensus was reached.

#### 3.4.2. Coding and Affinity Mapping

Using the diarised and merged transcript, an inductive thematic analysis was performed following the approach described by Clarke and Braun [47]. To support the generation of preliminary codes, the transcript was imported into ChatGPT (GPT-4.1) [46], consistent with emerging practices for LLM-assisted qualitative analysis [48,49]. The LLM output was used exclusively as a starting point to accelerate the initial coding phase and was never adopted without critical evaluation. The preliminary codes and their locations within the transcript were subsequently verified, reviewed, and refined by the researchers. The finalised codes were compiled into a codebook, listing each code alongside its frequency of appearance within the transcript.

Based on the compiled codebook, an affinity mapping was carried out to cluster conceptually related codes and to identify overarching thematic categories reflecting the shared concerns and design priorities of the participants. The resulting categories were grounded not only in participants’ verbal expressions but also in the collective concerns and needs expressed across different professional roles and teams.

Finally, the co-designed visualisation prototypes were cross-referenced with the inductive codes and the affinity map. Each prototype was aligned with its corresponding thematic category, ensuring that the participant-generated visual artifacts were interpreted within the same semantic structure as the verbal data. This multimodal approach, integrating both the diarised transcript and the physical co-design materials, provided a comprehensive basis for translating participant insights into visualisation requirements.

#### 3.4.3. Feasibility Assessment

Following the inductive coding, affinity mapping, and prototype cross-referencing, the identified occupational health themes and the discrete visualisation elements proposed by the two co-design groups were subjected to a systematic feasibility assessment. The purpose of this assessment was to determine which of the participant-generated concepts can be realised using the sensing configuration as defined in Table 2. Furthermore, it was identified which concepts require sensor modalities or data sources beyond the scope of the sensing platform.

The assessment was applied at two levels. At the thematic level, each inductive code derived from the workshop transcript was evaluated against the available data streams. At the prototype level, each discrete visualisation element proposed by either group was evaluated individually, given that prototype concepts are more concrete and may depend on multiple sensor modalities simultaneously.

Three classification categories were defined for both levels of analysis:

(1) **Directly addressable**: the theme or visualisation element can be measured or derived from data streams available within the current sensing platform, without requiring additional sensors, instruments, or data sources.
(2) **Partially addressable**: the theme or visualisation element can be approximated or partially realised using available data streams, but its full implementation requires one or more sensor modalities not currently available.
(3) **Out of scope**: the theme or visualisation element cannot be measured or approximated using the current sensing configuration.

The classification was applied by the research team based on direct inspection of the sensor capabilities listed in Table 2. Themes and prototype elements assigned to the partially addressable or out-of-scope categories are reported in the results alongside directly addressable ones. This was done to report genuine participant needs and constitute priorities for future extensions of the sensing platform and visualisation system.

### 3.5. Developed Data Visualisations

Based on the feasibility assessment, an initial set of data visualisations that integrated concepts from the co-designed prototypes was implemented in Python 3.12 using the matplotlib [50] and seaborn [51] libraries. The following visualisations were developed:

- **Physical activity and sedentary behaviour:** a daily activity timeline, an activity distribution comparison chart (sensor-derived vs. OSPAQ self-report [52]), and a daily step count and distance summary.
- **Heart rate:** a circular heart rate class distribution across the working week and a daily heart rate range chart.
- **Environmental noise exposure:** a noise exposure timeline and a daily noise distribution chart.
- **Postural load:** a multi-view postural load visualisation displaying trunk orientation in the superior, lateral, and posterior anatomical projections.

Following development, the visualisations were presented to the co-design workshop participants in a dedicated follow-up session. Participants were invited to provide structured feedback and propose refinements, which were subsequently incorporated into the final designs. This iterative review step ensured alignment between the implemented visualisations and stakeholder expectations.

## 4. Results

### 4.1. Narrative Synthesis of Co-Design Workshop

Inductive coding of the co-design workshop transcript yielded 17 distinct codes, which were subsequently clustered into five higher-order thematic categories through affinity mapping: *Stress*, *Posture*, *Physical Activity*, *Environmental Factors*, and *Data Visualisation and Awareness*. Table 3 presents the resulting affinity map, listing each code alongside its thematic category and frequency of appearance (f.a.) within the included participant utterances. Codes with higher frequencies reflect concerns raised recurrently across participants and across different stages of the discussion, and therefore carry greater weight as design requirements. The thematic narrative below follows the affinity map categories.

#### 4.1.1. Stress

Work-related stress was the most frequently coded theme (f.a. = 13). Participants described stress not as a static state but as a dynamic phenomenon that fluctuated across the workday. According to the participants, stress is driven by the nature and intensity of service interactions, organisational constraints, and environmental conditions. Customer service managers in front-office roles emphasised the near-continuous character of stress exposure. Interruptions were identified as a primary driver, with one participant noting that no service interaction in their workplace was completed without external disruption. Another participant described the influence of shared service floors, where noise from adjacent desks, waiting clients, and competing conversations was experienced as a sustained physiological burden. Instances of verbal aggression by members of the public toward service staff were also reported, underscoring the severity of the psychosocial risk context.

Noise distraction (f.a. = 6) was identified as both a stressor in its own right and a mediating factor that amplified the intensity of service interactions. Participants described the acoustic environment of shared front-desk settings as a significant source of cognitive load and difficulty concentrating.

Additionally, limited control of workers over their pauses was mentioned several times. Participants acknowledged that mandatory or self-initiated breaks were often structurally impossible in high-demand front-office settings. This raised the question of whether a notification system prompting breaks based on elapsed work time would be perceived as helpful or as an additional stressor. In that regard, one participant acknowledged that such prompts might increase rather than reduce distress in contexts where breaks are organisationally infeasible.

Work organisation (f.a. = 7), the third most frequently coded theme within the stress category, reflects the organisational and structural conditions under which work is performed. In particular, task allocation, rotation between front-office and back-office roles, the management of service queues, staffing levels, and the distribution of cognitively demanding tasks were pointed out. Participants identified these factors as primary drivers of occupational stress and musculoskeletal strain. Consequently, it was proposed that data-driven insights about physiological risk patterns could support evidence-based decisions about work scheduling and role rotation. One participant articulated this explicitly, describing the joint responsibility of workers, managers, and occupational health departments in translating risk data into organisational improvement measures.

#### 4.1.2. Posture

Postural risk and ergonomic concerns were the second most frequently coded theme (f.a. = 10). This theme was particularly mentioned by participants with occupational health and ergonomics backgrounds. Discussions covered trunk inclination, lateral asymmetries, and the distinction between seated and standing static postures. Participants emphasised the temporal dimension of postural risk. They were interested not only in instantaneous posture but in patterns across the workday, including how posture evolved relative to fatigue, break periods, and environmental conditions. Proposals for three-axis posture visualisations, combining views from above, from the side, and from behind, were put forward to capture the full spatial distribution of postural loading.

Muscular tension (f.a. = 5) was discussed extensively in relation to postural loading. In particular, the oscillation of tension levels across the workday and their dependence on task intensity were noted. Participants proposed body-region-specific comparisons, distinguishing between shoulder, neck, and spinal loading, and suggested correlating tension levels during active service periods with periods of rest or lower cognitive demand.

Pain symptom reporting (f.a. = 5) was raised as a necessary complement to objective sensor data. Participants expressed interest in tracking not only the physiological and postural correlates of discomfort but also the subjective experience of pain. This includes its location, intensity, and temporal relationship to specific tasks or environmental conditions. Proposals included body mapping tools that would allow workers to record where discomfort was felt and when. The correlation of such records with sensor-derived activity and posture data was an additional point of interest.

Work equipment (f.a. = 2) and formal ergonomic assessment (f.a. = 1) were raised in the context of the relationship between postural risk and workstation configuration. Configurations such as the chair type, desk height, monitor position, and lumbar support were mentioned, among others. Participants proposed integrating workstation variables alongside postural data to allow joint interpretation of equipment and posture.

#### 4.1.3. Physical Activity and Active Breaks

Active breaks (f.a. = 6) emerged as a high-priority theme. Participants requested explicitly tracking the duration of breaks, their frequency, and quality. The emphasis was not simply on the absence of sedentary behaviour but on the nature of the transition. This would mean whether a break involved genuine physical displacement or remained confined to the immediate workstation area.

Sedentary behaviour (f.a. = 2) and human activity recognition (f.a. = 2) were further noted. Participants mentioned that the ability to distinguish between sitting, standing, and walking states, and the quantification of their respective duration across the workday, would be beneficial. Participants proposed that break patterns be visualised alongside physiological indicators so that the relationship between recovery periods and stress or muscular load could be made explicit.

#### 4.1.4. Environmental Factors

Environmental stressors were a recurrent concern, with participants identifying noise distraction (Section 4.1.1), temperature discomfort (f.a. = 2), and light conditions (f.a. = 1) as factors influencing both physiological state and postural behaviour. Temperature discomfort was described as a factor that influenced both muscular tension and general well-being. Light conditions were mentioned as a driver for postural adjustment, given that insufficient or poorly directed lighting contributed to sustained awkward postures. The broader category of environmental conditions (f.a. = 2) encompassed both of these dimensions alongside general workspace comfort.

#### 4.1.5. Data Visualisation and Awareness

Although data visualisation (f.a. = 2), risk awareness (f.a. = 1), and sensor data (f.a. = 1) had relatively low frequencies, their qualitative impact was substantial. Participants valued representations that could synthesise physiological, contextual, and postural data into interpretable formats. These were pointed out as particularly useful if they could offer daily or weekly summaries with clear risk thresholds and actionable recommendations. The differentiation between worker-facing, manager-facing, and occupational health professional-facing views was a recurring theme. This reflects the need for role-adapted granularity in how risk information is communicated. The identified requirements informed the design priorities pursued in the co-design prototype session.

### 4.2. Co-Designed Visualisation Prototypes

**Figure 2:**
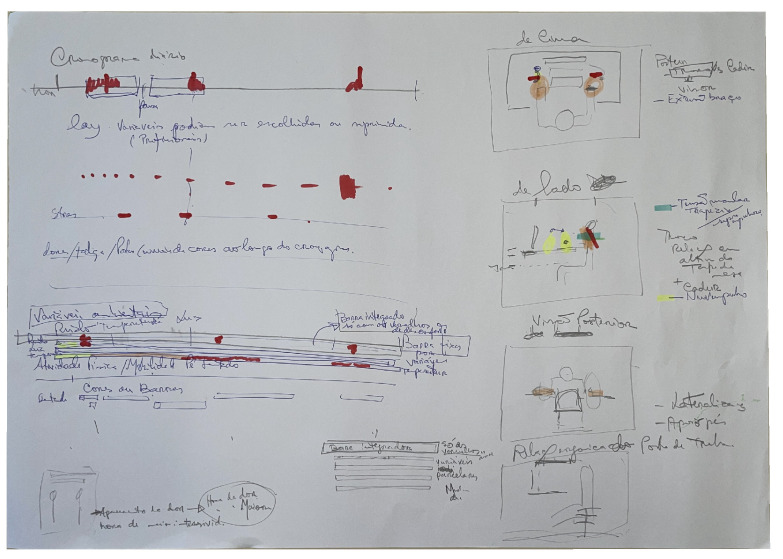
Low-fidelity visualisation prototype developed by Group 1 during the co-design workshop. The design centres on an integrative daily chronogram with layered variable tracks (**left**) and three complementary spatial posture views with a workstation ergonomic sketch (**right**).

**Figure 3:**
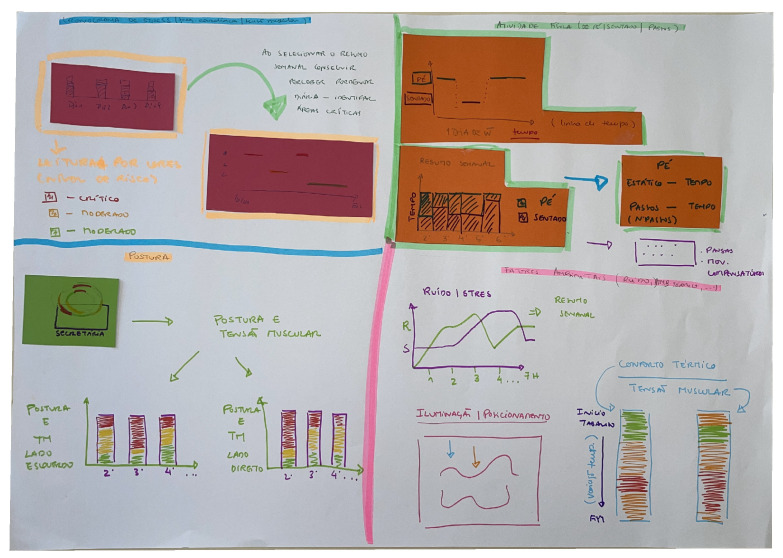
Low-fidelity visualisation prototypes developed by Group 2 during the co-design workshop. The sheet presents distinct visualisation concepts covering stress (**top-left**), physical activity (**top-right**), posture (**bottom-left**), and environmental factors (**bottom-right**), each with explicit weekly and daily views.

During the co-design workshop, participants were organised into two working groups, each tasked with developing low-fidelity visualisation prototypes on A2 paper. The groups presented their proposals at the end of the session, followed by a collective discussion. The results are presented in Figure 2 and Figure 3, respectively. While both groups addressed the same four thematic categories identified through affinity mapping (stress, posture, physical activity, and environmental factors), their design approaches differed substantially in underlying architecture and intended audience. It should be noted that the prototypes developed by each group comprise collections of distinct visualisation concepts rather than a single unified interface. The spatial organisation of each A2 sheet reflects the available paper area rather than a proposed screen layout. The first group developed a set of concepts anchored around an integrative daily chronogram, with layered, expert-configurable variable tracks and multi-axis postural representations, with an emphasis on professional-level analysis. The second group proposed a set of independent visualisation concepts covering stress, physical activity, posture, and environmental factors, each with explicit weekly and daily views. These were designed for use by both individual workers and occupational health professionals. The following subsections describe each group’s concepts in detail.

#### 4.2.1. Group 1: Integrative Daily Chronogram

The first group organised their prototype around a single horizontal daily timeline (*Cronograma diário*) representing the full workday from start to finish. Along this timeline, discrete events are plotted as colour-coded markers. Red markers indicate risk during work periods, and pauses are indicated in between boxed segments. The fundamental design principle underlying this prototype was the concept of configurable layers. The notation on the sketch states explicitly that variables could be chosen or toggled by professionals. Thus, the visualisation would be adaptable to different analytical contexts rather than fixed in content.

Below the timeline, the prototype adds two parallel horizontal tracks. The first plots stress as a separate track using the same axis. A note below both tracks reads *"dores/fadiga/postura—variáveis de cores ao longo do cronograma"* (pain/fatigue/posture—colour-coded variables along the chronogram), indicating that subjective discomfort states are intended to be represented using the same colour-along-time convention. The second plots environmental variables, such as noise (*ruído*), temperature (*temperatura*), and light (*luz*), allowing their co-occurrence with work and pause periods to be read directly.

A central structural element is the *Barra Integradora* (integrator bar), a composite summary bar positioned at a higher level of abstraction. This bar aggregates risk signals from all tracked variables into a single colour-coded ribbon. Sections of the integrator bar appearing in red indicate periods of combined discomfort or risk, regardless of which specific variable is responsible. The design includes an expandable mechanism. Selecting a red segment would expand the view to show the *variáveis parcelares* (component variables) contributing to that risk period. Each component then appears as a separate fixed sub-bar. This creates a three-level hierarchy consisting of the integrator bar, current variable set, and component variables. These were intended to serve different user roles at different levels of detail.

In addition to the timeline-based panels, the prototype incorporates a *pain annotation feature*. This feature consists of a timestamped log allowing the worker to record the time at which pain was experienced (*hora de dor*). This element acknowledges the irreducibly subjective nature of pain and proposes a lightweight self-report mechanism to complement objective measurements.

The right column of the prototype is devoted to postural representation through three distinct spatial views. The *top-down view* (*de Cima*) depicts the worker at a desk from above. The *lateral view* (*de Lado*) shows the worker in profile, with colour-coded markers identifying muscle tension at the trapezius muscles (labelled explicitly in cyan). Additionally, the thorax position relative to desk height and the corresponding position of the wrists (indicated in yellow) are depicted. Thus, this view integrates workstation variables, such as the desk surface height and chair configuration, with potential musculoskeletal tension markers. The *posterior view* (*Visão Posterior*) depicts the worker from behind and emphasises lateral trunk displacement, enabling the detection of asymmetric loading patterns. Finally, a fourth sketch proposes a dedicated *workstation ergonomic view* (*Relação Ergonómica do Posto de Trabalho*). This sketch depicts the desk from the side to document the spatial relationship of the utilised equipment, e.g., desk height, monitor position, and chair geometry.

#### 4.2.2. Group 2: Thematic Weekly–Daily Visualisation Concepts

The second group proposed a set of independent visualisation concepts organised thematically across their working sheet, with each concept addressing one of the four thematic categories. Each visualisation depicts the corresponding variables evolving either over the workday, the entire week, or both. Colour-coded risk levels are used to indicate critical, moderate, or low risk. These are rendered in red, yellow, and green, respectively. Thus, the visualisations proposed by this group share a common visual design language.

The stress panel (top-left) is headed by the label *“Cronograma de Stress (Freq. Cardíaca|Tensão Muscular)”*, identifying heart rate and muscular tension as the intended data sources. The weekly view consists of a grouped bar chart with one bar per day (Dia 1 through Dia 4 visible in the sketch). Each bar is subdivided into colour bands corresponding to the three risk levels. An annotation beside this chart reads: *“Ao selecionar o Resumo Semanal conseguir perceber ao pormenor—Diária—Identificar Áreas Críticas”* (By selecting the weekly summary, gain access to the daily detail—identify critical areas). This drill-down interaction is illustrated by a second panel that depicts a temporal distribution of the measured risk factors along the day. This view enables the identification of time periods in which each risk factor was observed.

The physical activity panel (top-right) is organised around two charts. The first depicts a single workday as a step-function chart with two states, standing (*Pé*) and seated (*Sentado*). These are plotted against a continuous time axis in the same fashion as in the stress panel. The second chart presents a weekly summary as a grouped bar chart, with one stacked bar per day showing the proportion of time spent standing and seated. An arrow from this chart leads to a detail box that decomposes standing time into two ergonomically distinct sub-states: *static standing* (time spent in a fixed standing position) and *walking* (number of steps taken). This distinction was explicitly motivated, as the group recognised that a step counter alone does not capture static standing. It was stated that occupational ergonomics treats static standing posture as a risk factor independent of step count. Numeric information about, for example, the number breaks and compensatory movements is also provided.

The posture quadrant (bottom-left) is introduced by an overhead map of the worker’s spatial position at the desk (labelled *Secretária*). This spatial displacement view is intended to show where the worker spent most of the time within the workstation area while seated. From this overhead map, two arrows lead to a pair of parallel time-series bar charts. The left chart is labelled *“Postura e TM—Lado Esquerdo”* (Posture and Muscular Tension—Left side) and the right *“Postura e TM—Lado Direito”* (Posture and Muscular Tension—Right side). Each chart plots stacked multi-coloured bars at daily intervals (2’: Monday, 3’: Tuesday, etc.) The bilateral structure of this visualisation is designed to make asymmetries in postural loading and muscular tension visible. Thus, this visualisation supports the identification of habitual compensatory postures that might not be apparent from single-side or aggregate measures.

Finally, the environmental factors panel (bottom-right) contains three linked visualisations. The first is a dual-line chart superimposing ambient noise (*R*) and stress (*S*) over a seven-hour workday axis. The intention behind this is to make the temporal co-variation of noise exposure and physiological stress immediately readable. The second visualisation addresses the relationship between lighting conditions and postural positioning (*Iluminação|Posicionamento*). This is depicted as a conceptual sketch showing undulating curves, with directional arrows suggesting that changes in ambient light drive postural adjustments. The third visualisation pairs *thermal comfort* (*Conforto Térmico*) with *muscular tension* (*Tensão Muscular*) in two vertically stacked strip charts aligned on a shared time axis, lasting the entire workday. Over the workday, thermal comfort and muscular tension are plotted as colour-coded strips, indicating that the values would be extracted for specific time windows. The design aims to reveal whether thermal discomfort and muscular tension covary across the workday.

#### 4.2.3. Cross-Cutting Observations from the Collective Discussion

Following the two group presentations, a collective discussion surfaced several additional design considerations that applied across both prototypes. One participant proposed adding a *work intensity indicator*, a daily count of service interactions, as a contextual complement to the physiological stress chronogram. The reasoning behind this being that correlating interaction volume with heart rate data would help distinguish organisationally driven stress from other sources. A related proposal was the inclusion of a *critical event flag*: a worker-initiated marker that could be placed manually on the timeline at moments of particular psychological or physical stress.

A further cross-cutting theme was the differentiation of visualisation complexity by user role. One participant observed explicitly that workers less accustomed to data-dense interfaces would benefit from simpler, more concrete representations.

Finally, participants converged on the behavioural purpose of these visualisations. The primary value of the system was not monitoring per se but the promotion of behaviour change across the organisation and its workers. Colour-coded risk indicators were explicitly valued as *“facilitating”* in this regard, making risk states legible without requiring technical literacy.

### 4.3. Feasibility Assessment

The participant-generated insights reported in Section 4.1 and Section 4.2 span a broader range of occupational health phenomena and visualisation concepts. Not all can be fully realised using the smartphone and smartwatch sensing configuration deployed in this study, as defined in Table 2. Following the feasibility assessment methodology described in Section 3.4.3, each inductive code and each discrete prototype element was classified as directly addressable, partially addressable, or out of scope. This section presents the results of this assessment and explains the reasoning underlying each classification. Particular attention is given to the partially addressable and out-of-scope items, as these represent priorities for future platform extensions.

#### 4.3.1. Theme-Level Feasibility

Table 4 presents the feasibility classification of all 17 inductive codes derived from the workshop transcript. Fourteen directly or partially addressable codes correspond to phenomena measurable through the available sensor streams. The remaining seven codes fall partially or entirely outside the current platform’s scope.

##### Partially Addressable Themes

Work organisation cannot be measured directly by any wearable sensor, as it reflects organisational and structural conditions (e.g., task allocation, staffing levels, queue management, and role rotation) rather than a physiological or behavioural signal. However, the downstream physiological and behavioural consequences of poor work organisation, namely elevated heart rate, prolonged uninterrupted sedentary periods, and the absence of break transitions, are detectable through the smartwatch and smartphone IMU, respectively.

Muscular tension requires surface electromyography (EMG) to directly quantify muscle activity. While the smartphone IMU provides trunk inclination and lateral tilt data that can indicate awkward or sustained static postures, these do not constitute a direct tension measure and should not be treated as equivalent.

Ergonomic assessment and environmental conditions are partially addressable because IMU-derived postural data can serve as objective supporting evidence within a broader ergonomic evaluation, and ambient noise is directly captured by the smartphone microphone.

##### Out-of-Scope Themes

Pain and symptom reporting is inherently subjective and cannot be inferred from any sensor modality available in the current platform. While correlations between postural load and pain onset are well established in the musculoskeletal literature [53,54,55,56], sensor data alone cannot validate or quantify a subjective symptom. Integration of structured self-report instruments, such as digital body maps or numeric pain rating scales, would be required.

Work equipment and workstation ergonomics cannot be characterised from inertial data alone. Sensor data may reflect the postural consequences of a suboptimal workstation setup, but a formal workstation assessment addressing desk height, chair configuration, monitor position, and foot support requires structured instruments such as the ROSA [39].

Temperature discomfort and light conditions require dedicated environmental sensors such as thermometers or humidity sensors and lux meters, respectively. These are not part of the current smartphone or smartwatch configuration as defined in Table 2.

#### 4.3.2. Prototype-Level Feasibility

Table 5 presents the feasibility classification of all 27 discrete visualisation elements proposed across the two co-design groups and the collective discussion. The reasoning for partially addressable and out-of-scope classifications at the prototype level reflects and extends the theme-level analysis above, with two additional elements arising from the greater specificity of the prototype concepts.

The *stress chronogram* proposed by Group 2 was explicitly designed to integrate both heart rate and muscular tension. Heart rate is directly available, but muscular tension requires EMG, which is not part of the current platform. The chronogram can therefore be implemented using heart rate as the sole physiological stress proxy, with EMG integration deferred to future system extensions.

### 4.4. Developed Data Visualisation

The visualisations presented in this section are based on real-world data collected from office workers participating in the PrevOccupAI+ project. The examples shown are drawn from representative monitoring periods and were selected to illustrate the informational content and interpretive potential of each visualisation type. Where applicable, the observed data patterns are briefly discussed in terms of their occupational health implications for the individual worker, reflecting the intended use of the visualisations as tools for self-monitoring and structured reflection.

#### 4.4.1. Physical Activity and Sedentary Behaviour

##### Daily Activity Timeline

**Figure 4:**
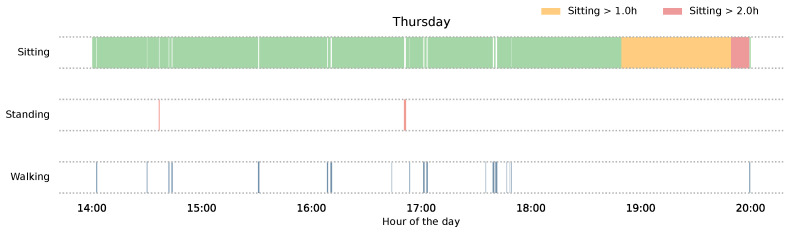
Daily activity timeline for a representative workday (Thursday). Each horizontal bar encodes a distinct activity state throughout the shift: seated (green), standing (salmon), and walking (blue). Within the seated bar, the colour transitions to yellow following one hour of uninterrupted sitting and to red upon exceeding two hours of continuous sedentary time.

Figure 4 presents the daily activity timeline for a representative workday (Thursday). This timeline has been extracted through a human activity recognition model as described in [57], with some additional post-processing steps. Each of the three horizontal bars encodes a distinct activity state across the shift: seated (green), standing (salmon), and walking (blue). Within the seated bar, the colour progressively transitions to yellow following one hour of uninterrupted sitting, and to red upon exceeding two hours of continuous sedentary time without interruption. This feature, indicating risk of prolonged sedentary behaviour, was proposed during the follow-up session.

On the day illustrated, the participant was predominantly seated throughout the shift, with only brief and infrequent standing and walking episodes. A sustained sedentary bout is visible in the late afternoon, during which the seated bar transitions first to yellow and subsequently to red, indicating that the participant remained seated continuously for more than two hours without a postural break.

##### Activity Distribution

**Figure 5:**
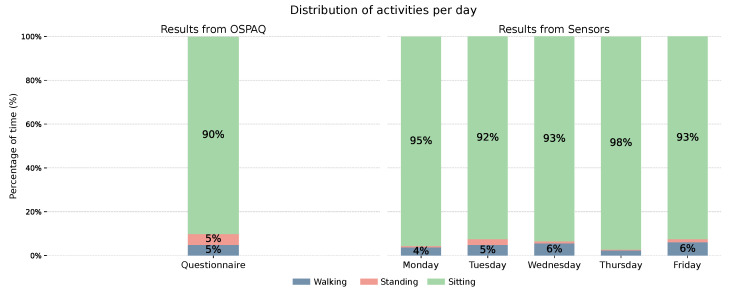
Activity distribution across the monitored working week. The leftmost bar represents the participant’s self-reported estimate of time spent seated, standing, and walking, as obtained from the OSPAQ questionnaire. The remaining bars display the corresponding sensor-derived distributions for each monitored workday. Proportions below 2% are omitted for legibility.

Figure 5 presents the activity distribution across the monitored working week. The leftmost bar reflects the participant’s self-reported activity distribution as collected through the usage of the OSPAQ questionnaire [52]. The remaining bars display the sensor-derived distributions for each monitored workday. Proportions below 2% are omitted from the bars for legibility. The juxtaposition of self-reported and sensor-derived estimates was deliberately designed to prompt reflection on the discrepancy between perceived and objective activity patterns.

Across all days, the sensor data consistently indicate a predominantly sedentary work pattern, with seated time ranging from 92% to 98% of the shift, and combined walking and standing time not exceeding 8% on any given day. The self-reported estimate was broadly consistent with the sensor-derived results, with the participant reporting 90% seated time, 5% standing, and 5% walking.

##### Daily Step Count

**Figure 6:**
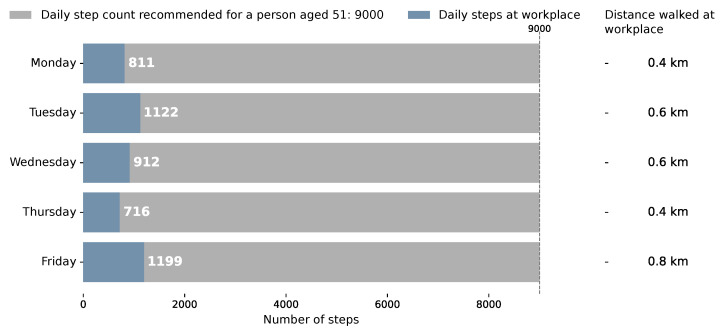
Daily workplace step count and distance covered across the monitored working week. The blue portion of each bar indicates the number of steps recorded during the work shift; the grey portion represents the gap relative to the age-adjusted recommended daily step target (9000 steps for a person aged 51 years). The distance covered within the workplace on each day is annotated to the right of each bar.

Figure 6 presents the daily step count and distance covered within the workplace across the monitored week. For each day, the blue portion of the bar represents the number of steps recorded during the work shift. The grey portion represents the remaining steps needed to reach the age-adjusted recommended daily target [58], which is indicated at the top of the chart. The corresponding distance covered during working hours is annotated.

Across the monitored week, the participant accumulated between 716 and 1199 steps per workday, covering distances ranging from 0.4 km to 0.8 km. In all cases, workplace step counts fell substantially short of the recommended daily target of 9000 steps for a person of 51 years of age [58]. The highest single-day count (1199 steps), representing approximately 13% of the recommended total, was achieved on Friday. This visualisation contextualises walking patterns during working hours against an individualised health benchmark, reinforcing the picture of a highly sedentary occupational profile observed in Figure 4 and Figure 5.

#### 4.4.2. Heart Rate

##### Circular Heart Rate Class Distributions

**Figure 7:**
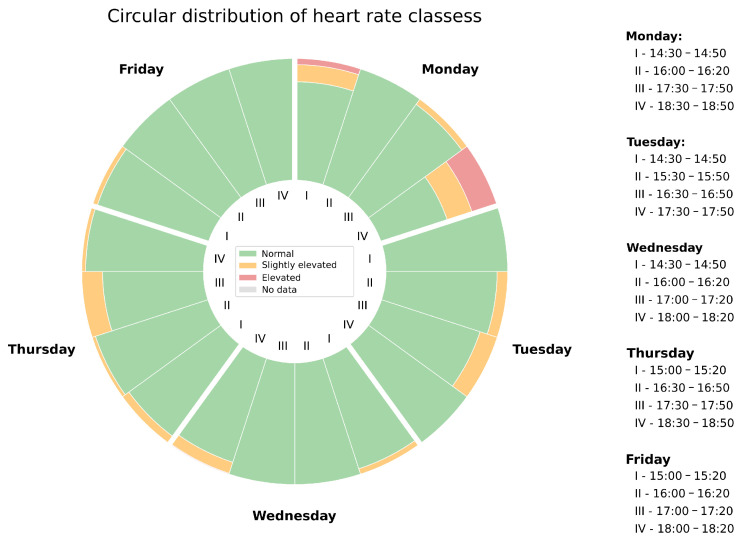
Circular distribution of heart rate classifications across the monitored working week. Each sector corresponds to one workday, subdivided into four 20 min acquisition sessions whose time intervals are listed to the right. Arc segments are colour-coded according to three classification levels based on the heart rate ratio: normal (green, ratio <30%), slightly elevated (yellow, 30–39%), and elevated (red, >39%).

Figure 7 presents the weekly circular distribution of heart rate classifications across the monitored working week during instances in which the participant was seated (i.e., at their workstation). To enable meaningful comparison across individuals, heart rate was expressed by the heart rate ratio [59]. Based on this ratio, three classification levels were defined: *normal* (ratio below 30%), *slightly elevated* (ratio between 30% and 39%), and *elevated* (ratio above 39%). The rationale for these thresholds is that ratios between 30% and 39% are considered typical during light physical activity [60]. The presence of such values during sedentary work may therefore be interpreted as a potential indicator of occupational stress. Each day of the working week is represented as a sector of the chart, subdivided into four 20 min acquisition sessions whose corresponding time intervals are listed to the right of the figure. Arc segments are colour-coded according to the classification level.

Across the monitored week, the majority of acquisition windows were classified as normal. However, elevated and slightly elevated values were observed throughout the week, with Monday and Thursday having more prominent occurrences, suggesting transient periods of elevated cardiovascular demand during those workdays.

##### Heart Rate Range Chart

**Figure 8:**
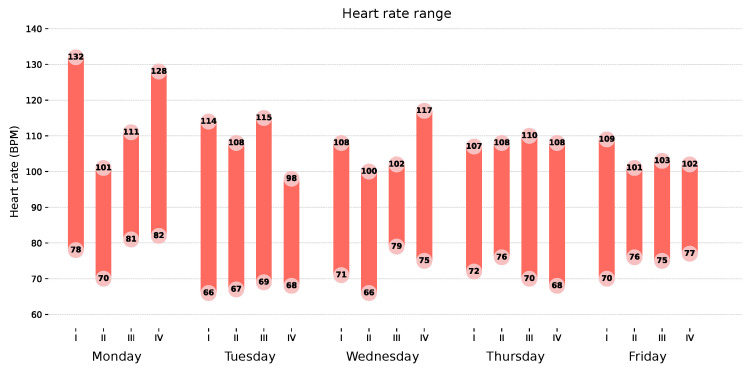
Heart rate range per acquisition window (I–IV) across the monitored working week. Each bar spans the minimum (base) to maximum (top) heart rate recorded during the corresponding 20 min window. The measured heart rate values are annotated directly on the bar.

Figure 8 presents the heart rate range recorded during each acquisition window (I–IV) across the monitored working week. Each bar spans the minimum to maximum heart rate observed within the corresponding 20 min window, with both values annotated directly on the bar. This layout enables comparison of within-day cardiovascular variability across acquisition windows, as well as between-day differences in overall heart rate range.

Across the monitored week, maximum heart rate values were consistently elevated, frequently exceeding 100 BPM, with the highest recorded values observed on Monday, namely session I with 132 BPM and session IV with 128 BPM. This is consistent with the elevated classifications identified in Figure 7. Minimum values remained relatively stable across the week, ranging from 66 to 82 BPM. The within-window ranges were generally broad, indicating considerable short-term heart rate variability during each acquisition period.

#### 4.4.3. Environmental Noise Exposure

##### NoiseExposure Timeline

**Figure 9:**
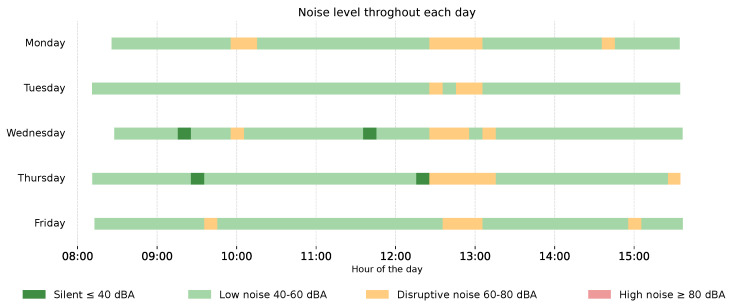
Noise exposure timeline across the monitored working week. Each horizontal bar corresponds to one workday, colour-coded according to four noise categories: silent (≤40 dBA, dark green), low noise (40–60 dBA, light green), disruptive noise (60–80 dBA, amber), and high noise (≥80 dBA, red).

Figure 9 presents the noise exposure timeline across the monitored working week. Each horizontal bar corresponds to one workday, with the horizontal axis representing the time of day. Noise levels are encoded using four colour categories: silent (≤40 dBA, dark green), low noise (40–60 dBA, light green), disruptive noise (60–80 dBA, yellow), and high noise (≥80 dBA, red). These categories were defined in consultation with the occupational health specialists during the follow-up co-design session. To construct the timeline, noise data were aggregated into 10 min intervals, with each interval assigned the most frequently occurring noise level within that period. Prior to data collection, the smartphone microphone was calibrated against a silent room reference to establish a baseline offset. It is important to note that these levels are meant to be seen as estimates of the true values. Measurement of noise levels using smartphones is not truly accurate. However, the presented data can still be informative and should be followed up by a true measurement using dedicated equipment if higher values are consistently measured.

Across all monitored days, low noise levels predominated, with disruptive noise episodes occurring intermittently throughout the shift. Silent periods were observed on Wednesday and Thursday, appearing as brief dark green segments. Disruptive noise was most sustained on Thursday, where an extended yellow segment is visible between approximately 12:30 h and 13:30 h. No instances of high noise were recorded across the monitored week.

##### Daily Noise Distribution

**Figure 10:**
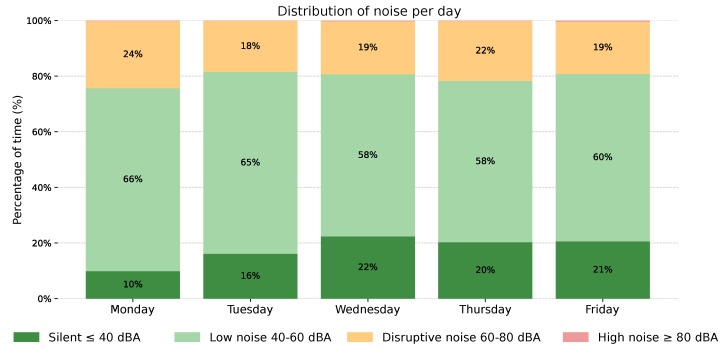
Daily noise exposure distribution across the monitored working week. Each stacked bar represents the proportion of shift time spent in each of the four noise categories: silent (≤40 dBA, dark green), low noise (40–60 dBA, light green), disruptive noise (60–80 dBA, yellow), and high noise (≥80 dBA, red).

Figure 10 presents the daily noise exposure distribution across the monitored working week. Each stacked bar corresponds to one workday and displays the proportion of shift time spent in each of the four noise categories.

Across all days, low noise (40–60 dBA) was the dominant exposure level, accounting for 58% to 66% of the shift. Disruptive noise (60–80 dBA) represented a consistent and non-negligible proportion of each workday, ranging from 18% on Tuesday to 24% on Monday. Silent periods (≤40 dBA) were most prevalent on Wednesday, Thursday, and Friday, where they accounted for approximately 20–22% of the shift, compared to 10% on Monday. No high noise (≥80 dBA) was recorded on any monitored day. Taken together with Figure 9, this visualisation provides a complementary aggregate perspective on the acoustic environment, enabling identification of days with a comparatively higher disruptive noise burden.

#### 4.4.4. Postural Load

**Figure 11:**
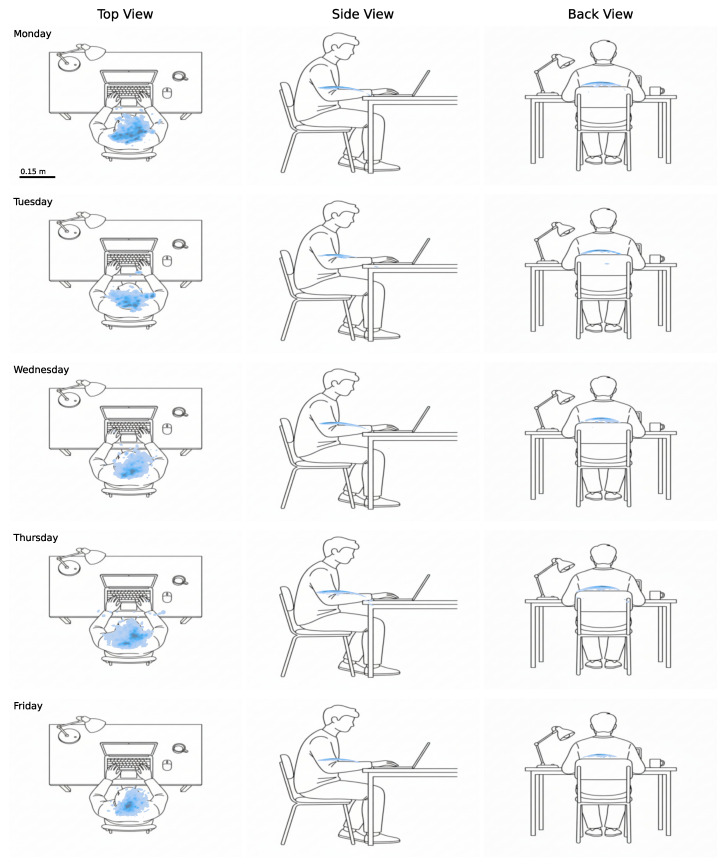
Multi-view postural load visualisation across the monitored working week. Each row corresponds to one workday; each column represents a distinct anatomical projection: top view (**left**), side view (**centre**), and back view (**right**). The blue density cloud represents the distribution of trunk positions recorded during seated periods, with darker regions indicating more frequently adopted postures.

Figure 11 presents the distribution of trunk posture during seated periods across the monitored working week. Each row corresponds to one workday, and each column represents a distinct anatomical projection: top view (left), side view (centre), and back view (right). The background schematic illustration is adapted to the participant’s sex. The density of recorded trunk positions is rendered as a two-dimensional KDE (kernel density estimation), with darker regions indicating positions adopted more frequently and lighter regions corresponding to positions occupied more briefly. The spread and intensity of the density cloud thus reflect both the range and the dwell time of postural variation throughout the day.

The top view provides a combined representation of forward/backward and lateral trunk displacement, offering an overview of overall postural variability within the horizontal plane. The lateral view isolates sagittal plane motion, capturing forward and backward trunk inclination and reflecting natural adjustments such as leaning toward the workstation or returning to an upright position. The posterior view represents lateral trunk deviation in the coronal plane, enabling identification of postural asymmetries that may indicate a habitual lateral bias during seated work.

Across all monitored days, the density clouds in the lateral and posterior views are concentrated in a narrow region, suggesting relatively consistent trunk positioning with limited sagittal and lateral movement. The superior view reveals slightly greater day-to-day variability in the combined postural distribution, most notably on Monday and Thursday, where the density cloud appears more dispersed and displaced relative to the remaining days.

## 5. Discussion

The present study applied a generative co-design framework [28] to elicit, structure, and translate occupational health visualisation requirements from a multi-stakeholder group of office workers and occupational health professionals. Subsequently, a set of visualisations grounded in the identified requirements using real-world data collected from a smartwatch and a smartphone was implemented. The following sections interpret the findings of each phase of this process and position them within the broader context of occupational health monitoring and participatory design.

### 5.1. Suitability of the Generative Co-Design Framework

The adoption of the generative co-design framework [28] proved appropriate for the occupational health visualisation context. The framework’s explicit separation of pre-design, co-design, and post-design phases supported a structured progression from contextual understanding to prototype elicitation and, ultimately, to requirement translation. The field study and questionnaire administered during the pre-design phase ensured that the subsequent workshop was grounded in the actual working conditions of the participant population, rather than in abstract or hypothetical occupational scenarios.

The multi-stakeholder composition of the co-design workshop, comprising office workers alongside occupational health specialists and ergonomists, contributed directly to the quality and diversity of the established requirements. Office workers brought experiential knowledge of daily working conditions, recurring discomforts, and perceived risk factors. On the other hand, health professionals contributed domain expertise on clinically relevant occupational exposure thresholds, ergonomic risk frameworks, and visualisation needs from a professional assessment perspective.

The follow-up session conducted after the initial implementation further validated the utility of the framework’s iterative structure. Participants reported that their expectations from the co-design workshop had been met and expressed that the developed visualisations, given their clear visual design and interpretability, could effectively support office workers in identifying occupational health risks. The gathered feedback resulted in the refinement of specific visualisation elements. For example, the noise exposure categories were finalised in consultation with occupational health ergonomists, and the sedentary risk colour-transition feature was introduced at this stage. These iterative refinements illustrate the added value of including a structured post-design evaluation loop and highlight the importance of stakeholder validation before concluding the implementation process.

### 5.2. Stakeholder-Driven Theme Identification and Feasibility

The thematic analysis of the co-design workshop transcript yielded 17 inductive codes, reflecting the diversity of occupational health concerns perceived as relevant by the participant group. The most frequently appearing themes broadly correspond to well-established risk domains in office ergonomics and WRMD research [2,33,53,55]. Their recurrence across both co-design groups and the collective discussion suggests that these represent primary and shared concerns among the participant population.

The prominence of work organisation as a theme is particularly noteworthy. It reflects an awareness among participants that physiological and postural risks do not arise in isolation but are mediated by structural working conditions such as task allocation, staffing levels, and the availability of break opportunities. This perception aligns with the occupational health literature, which has increasingly recognised organisational factors as upstream determinants of ergonomic and psychosocial risk [2,4,8]. From a technical perspective, however, work organisation cannot be directly measured by any sensor modality available in the current platform, as it constitutes an organisational construct rather than a physiological or behavioural signal. This disconnect between what participants identified as a risk factor and what the current system can observe underscores a fundamental challenge in sensor-based occupational health monitoring. In particular, the dominant risks identified by workers cannot always be covered through the deployment of sensorised systems alone.

The presence of out-of-scope themes, such as pain and symptom reporting, work equipment and workstation ergonomics, temperature discomfort, and light conditions, further illustrates this gap. While these themes represent legitimate occupational health concerns, they either require subjective self-report instruments, dedicated environmental sensors, or formal ergonomic assessment tools that lie beyond the smartphone and smartwatch platform deployed in this study. Their emergence from the co-design process is nonetheless informative. They allow for mapping the boundaries between what the current system can offer and what a more comprehensive occupational health monitoring platform would require, thereby providing a structured basis for prioritising future system extensions. Conversely, the directly and partially addressable themes, which together account for the large majority of the identified codes, confirm that the smartphone and smartwatch sensing configuration is sufficient to address the primary occupational health concerns expressed by participants and to support the implementation of the core visualisation components.

### 5.3. Visualisations as Communication Tools Between Stakeholders

A recurrent theme across both co-design groups and the collective discussion was that the primary value of the proposed system lay not in automated monitoring per se, but in its capacity to support meaningful communication among workers, occupational health professionals, and organisational decision-makers. The effective use of these visualisations as communication tools requires that both the worker and the health professional share a common understanding of what the data represent. The multi-stakeholder composition of the co-design process ensured that the resulting designs were interpretable to both groups. The confirmation of visualisation clarity during the follow-up session further supports the conclusion that the proposed designs fulfil this communicative purpose.

The role-differentiated view complexity proposed during the collective discussion reflects an important design consideration: workers less accustomed to data-dense interfaces require simpler, more concrete representations, while occupational health professionals may benefit from richer, more granular views. The visualisations developed in the present study were intentionally designed to prioritise clarity and direct interpretability, with colour-coded risk indicators valued explicitly by participants as facilitating legibility without requiring technical literacy. This positions the current implementation primarily as a worker-facing tool that is simultaneously accessible to health professionals, thereby serving as a shared reference point for structured occupational health dialogue.

### 5.4. Limitations

Several limitations of the present study warrant acknowledgement. First, the co-design workshop involved a relatively small number of participants recruited from a single public administration organisation, which limits the generalisability of the identified themes and design requirements to other occupational sectors or cultural contexts. Future co-design studies should aim to include larger and more occupationally diverse participant groups to increase the breadth of the resulting requirements.

Second, the primary focus of the present study is the co-design process itself, as well as the development of the presented occupational health visualisations. Their implementation is demonstrated using real-world data collected from representative office workers. However, a population-level quantitative assessment of these still has to be carried out as a priority of future work. This assessment should encompass the evaluation of usability, interpretability, and impact on occupational health behaviour.

Third, the current sensing platform is limited to the modalities available on a consumer-grade Android smartphone and smartwatch. This constrains the range of occupational health phenomena that can be directly observed. For example, the absence of EMG means that muscular tension, a factor highlighted repeatedly by participants, can only be inferred indirectly from postural proxy measures derived from the IMU. Additionally, the battery capacity of the smartwatch imposes constraints on continuous data acquisition: heart rate data are currently collected across four discrete 20 min sessions per shift rather than continuously, which may result in the under-detection of transient physiological events occurring between acquisition periods.

Finally, the data streams derived from consumer-grade sensors cannot be considered equivalent to measurements obtained with dedicated, properly calibrated instrumentation. This is particularly relevant for the smartphone microphone-based noise assessment, for which the reported dBA values represent estimates of the ambient acoustic environment rather than accurate measurements. Consumer-grade microphones are not designed for precision sound level measurement, and their output is subject to device-specific frequency responses and placement variability. The resulting estimates should therefore be interpreted as general indicators of the acoustic exposure profile, suitable for identifying periods and patterns of elevated noise that may warrant further investigation, rather than as definitive exposure values. Cases in which consistently elevated noise levels are identified should be followed up with dedicated and properly calibrated sound level measurement equipment.

### 5.5. Future Work

Several directions for future development emerge from the present study. The interactive design elements proposed by the first co-design group, such as the configurable variable layer toggles and drill-down mechanisms enabling navigation from weekly summaries to daily detail, were not implemented in the current visualisation framework. These represent questions of application implementation rather than data availability. Their development would substantially increase the functional utility of the system for end-users. Similarly, the critical event flag and the role-differentiated view complexity proposed during the collective discussion could be addressed through application-level design in future iterations.

The partially addressable themes identified during the feasibility assessment highlight two priority extensions at the sensing level. Integration of EMG would enable direct quantification of muscular tension, completing the stress chronogram and postural load visualisations as originally envisaged by the co-design groups. Expansion of the sensing platform to include dedicated environmental sensors (e.g., lux meters and thermometers) would address the lighting and thermal comfort conditions currently classified as out of scope, closing the remaining gap between the occupational health concerns expressed by participants and the phenomena observable by the system.

The extension of the individual-level visualisation framework to support population-level reference values and aggregate dashboards would substantially broaden the utility of the system for organisational occupational health surveillance, enabling institutional decision-makers to monitor workforce-level exposure trends alongside individual worker profiles.

Finally, this study presented only a qualitative evaluation of the developed visualisations through the described follow-up session. A study that quantitatively evaluates the presented visualisations in terms of usability, interpretability, and short- as well as long-term impact on occupational health behaviour needs to be carried out in the future.

## 6. Conclusions

This study applied a generative co-design framework to develop occupational health visualisations grounded in the requirements of a multi-stakeholder group comprising office workers and occupational health professionals. The structured progression through pre-design, co-design, and post-design phases enabled the systematic elicitation of 17 occupational health themes and the assessment of their technical feasibility relative to a smartphone and smartwatch sensing platform. Furthermore, it allowed for the subsequent implementation of a set of visualisations addressing physical activity and sedentary behaviour, heart rate, environmental noise exposure, and postural load.

The co-design process confirmed that the adopted framework is well-suited to the occupational health visualisation context, facilitating meaningful dialogue between experiential and domain-expert knowledge. The feasibility analysis demonstrated that the smartphone and smartwatch configuration is sufficient to address the majority of the identified occupational health concerns directly or partially, while simultaneously delineating the boundaries of the current platform and identifying clear directions for future system extension. The follow-up session qualitatively validated the interpretability of the developed visualisations and led to targeted refinements.

Future work should prioritise the formal evaluation of the developed visualisations with a broader and more occupationally diverse user population, with particular attention to usability, behaviour change support, and the integration of interactive application-level features proposed during the co-design workshop. Expansion of the sensing platform through the addition of surface electromyography and dedicated environmental sensors would further close the gap between the occupational health concerns expressed by participants and the phenomena currently observable by the system.

## Figures and Tables

**Table 1:** Co-design workshop participants.

ID	Role	Gender	YE-CML
001	Ergonomist	F	7
002	Specialist in Occupational Safety	M	3
003	Specialist in Occupational Health	F	2
004	Specialist in Occupational Health	F	20
005	Customer Service Manager	F	5
006	Customer Service Manager	F	18
007	Customer Service Manager	F	22
008	Customer Service Manager	F	30
009	Customer Service Manager	F	30
010	Psychologist	F	24
011	Psychologist	F	30
012	Health Professional	M	25

YE-CML: years of experience within CML.

**Table 2:** Information extractable from the utilised devices, as presented to co-design workshop participants.

Device	Sensor	Extractable Information
Smartphone	Accelerometer Gyroscope Magnetometer Rotation Vector	Human activities (e.g., sitting, standing, and walking) Upper body movement (e.g., trunk displacement when seated) General spatial orientation of the worker
Smartphone	Internal Microphone	Approximate ambient noise levels (dBA)
Smartwatch	Accelerometer Heart Rate	Wrist movements (e.g., acceleration and repetitive movements) Heart rate (beats per minute)

**Table 3:** Affinity map derived from inductive coding of the co-design workshop transcript. Codes are clustered into higher-order thematic categories; frequency of appearance (f.a.) reflects counts within included participant utterances only.

Category	Code	f.a.
Stress	Work stress	13
	Work organisation	7
	Noise distraction	6
Posture	Ergonomics/posture	10
	Muscular tension	5
	Pain/symptom reporting	5
	Work equipment	2
	Ergonomic assessment	1
Physical Activity	Active breaks	6
	Sedentary behaviour	2
	Human activity	2
Environmental Factors	Environment conditions	2
	Temperature discomfort	2
	Light conditions	1
Data Visualisation & Awareness	Data visualisation	2
	Risk awareness	1
	Sensor data	1

f.a.: frequency of appearance

**Table 4:** Feasibility classification of inductive codes relative to the smartphone and smartwatch sensing configuration defined in Table 2.

Code	Feasibility	Available Sensor(s)/Rationale
Work stress	Directly addressable	Smartwatch HR
Work organisation	Partially addressable	Physiological consequences via Smartwatch HR and Smartphone IMU; organisational construct not sensor-measurable
Noise distraction	Directly addressable	Smartphone microphone
Ergonomics/posture	Directly addressable	Smartphone IMU
Muscular tension	Partially addressable	Postural proxy via Smartphone IMU; direct measurement requires EMG
Pain/symptom reporting	Out of scope	Subjective; requires self-report instrument
Work equipment	Out of scope	Requires ergonomic assessment instrument
Ergonomic assessment	Partially addressable	IMU-derived posture as supporting evidence; formal assessment requires ROSA or equivalent
Active breaks	Directly addressable	Smartphone IMU (activity recognition)
Sedentary behaviour	Directly addressable	Smartphone IMU
Human activity	Directly addressable	Smartphone IMU
Environmental conditions	Partially addressable	Noise via smartphone microphone; temperature and light require dedicated sensors
Temperature discomfort	Out of scope	Requires dedicated temperature/humidity sensor
Light conditions	Out of scope	Requires dedicated lux meter
Data visualisation	N/A—design requirement	Informs visualisation design; not a sensor-measurable phenomenon
Risk awareness	Directly addressable	Visualisation output derived from available sensor data
Sensor data	N/A—methodological reference	General discussion of sensor data; not a specific measurable phenomenon

HR: heart rate; IMU: inertial measurement unit (accelerometer, gyroscope, magnetometer); EMG: electromyography; f.a.: frequency of appearance.

**Table 5:** Feasibility classification of prototype visualisation elements relative to the smartphone and smartwatch sensing configuration defined in Table 2.

Group	Prototype Element	Available Sensor(s)	Feasibility
G1	Daily chronogram—work/pause timeline	Smartphone IMU	Directly addressable
G1	Configurable variable layer toggle	UI feature	Directly addressable
G1	Stress track (colour-coded events)	Smartwatch HR	Directly addressable
G1	Environmental track—noise	Smartphone microphone	Directly addressable
G1	Physical activity/mobility track	Smartphone IMU	Directly addressable
G1	Integrator bar with drill-down	Derived from all feasible streams	Directly addressable
G1	Posture view—posterior (lateralisation)	Smartphone IMU	Directly addressable
G1	Posture view—top-down (trunk, head, arm)	Smartphone IMU (trunk only)	Partially addressable
G1	Posture view—lateral (posture + muscle tension)	Smartphone IMU; EMG required for tension	Partially addressable
G1	Environmental track—temperature	None	Out of scope
G1	Environmental track—light	None	Out of scope
G1	Pain/discomfort annotation (timestamped)	Self-report input (manual)	Out of scope
G1	Workstation ergonomic view	Ergonomic assessment instrument	Out of scope
G2	Stress chronogram—daily drill-down	Smartwatch HR	Directly addressable
G2	Physical activity—daily step chart (*Pé*/Sentado)	Smartphone IMU	Directly addressable
G2	Physical activity—weekly grouped bar chart	Smartphone IMU	Directly addressable
G2	Physical activity—static standing vs. walking	Smartphone IMU; Smartwatch ACC	Directly addressable
G2	Breaks and compensatory movement panel	Smartphone IMU; UI feature	Directly addressable
G2	Posture—displacement map	Smartphone IMU	Directly addressable
G2	Environmental—noise vs. stress dual-line chart	Smartphone microphone; Smartwatch HR	Directly addressable
G2	Stress chronogram—weekly bar chart	Smartwatch HR; EMG deferred	Partially addressable
G2	Posture—bilateral bar charts (posture + TM)	Smartphone IMU; EMG deferred for TM	Partially addressable
G2	Environmental—lighting vs. positioning sketch	Dedicated light sensor required	Out of scope
G2	Environmental—thermal comfort vs. TM charts	Temperature sensor; EMG required	Out of scope
Both	Role-differentiated view complexity	UI feature	Directly addressable
Both	Critical event flag (worker-initiated)	UI feature (manual input)	Directly addressable
Both	Work intensity indicator (service count)	Administrative data source	Out of scope

TM: muscular tension; IMU: inertial measurement unit (accelerometer, gyroscope, magnetometer, rotation vector); HR: heart rate; ACC: accelerometer; UI: user interface.

## Data Availability

The original contributions presented in this study are included in the article. Further inquiries can be directed to the corresponding author.

## Abbreviations

The following abbreviations are used in this manuscript:

WRDs	Work-Related Disorders
WRMDs	Work-Related Musculoskeletal Disorders
CML	Câmara Municipal de Lisboa
ROSA	Rapid Office Strain Assessment
IMUs	Inertial Measurement Units
JITAIs	Just-In-Time Interventions
ASR	Automatic Speech Recognition
LLM	Large Language Model
f.a.	frequency of appearance
HR	Heart Rate
EMG	Electromyography

## Appendix A

### Appendix A.1. Co-Design Survey Questionnaire

**Table A1:** Summary of translated co-design survey questions and answer types.

ID	Section	Question	Answer Type/Options
Q1	General Information	What is your job or profession?	Multiple choice: (1) Office worker (2) Health professional (3) Specialist in Occupational Health (4) Psychologist (5) Other—option to specify
Q2	General Information	How long have you worked in your current profession within CML?	Numeric: (number of years)
Q3	General Information	How often do you consult health data?	Multiple choice: (1) Never (2) Once a year (3) Every six months (4) Once a month (5) Once a week (6) Daily
Q4	General Information	Where do you usually get health information?	Multiple choice: (1) Workplace reports (2) Health apps (3) Wearables (e.g., smartwatch, fitness tracker) (4) Medical appointments (5) Other—option to specify
Q5	General Information	If you use health or fitness apps, what types of data do you monitor?	Multiple choice: (1) Heart rate (2) Physical activity (3) Sleep patterns (4) Stress indicators (5) Movement/posture (6) Other—option to specify
Q6	Current Challenges and Needs	How easy or difficult is it for you to interpret health data when you receive it?	Likert Scale: (1) Very difficult (2) Difficult (3) Neutral (4) Easy (5) Very easy
Q7	Current Challenges and Needs	What challenges do you face when trying to understand or use this data?	Open answer
Q8	Current Challenges and Needs	When you look at health data visualisations (e.g., graphs, trend lines), what do you usually find confusing?	Multiple choice: (1) Too much information (2) Lack of explanation (3) Confusing visual elements (4) Hard to relate the visuals to my situation (5) Other—option to specify
Q9	Current Challenges and Needs	If you could improve the way health data is presented, what would you change?	Open answer
Q10	Data Interpretation and Visualisation Preferences	What type of data visualisation do you find easier to understand?	Multiple choice: (1) Charts (e.g., bar charts, line graphs, etc.) (2) Colour-coded risk levels (3) Simplified numerical values with explanation (4) Tables (5) Interactive data panels (6) Other—option to specify
Q11	Data Interpretation and Visualisation Preferences	If you’ve used a health or fitness app, which of the presented visualisations have you found most useful?	Open answer
Q12	Data Interpretation and Visualisation Preferences	Would you prefer to receive this type of information in real time (e.g., live updates) or in periodic summaries (e.g., daily or weekly reports)? Please explain your choice	Open answer
Q13	Data Interpretation and Visualisation Preferences	What factors would make visualisations of occupational health data easier for you to understand?	Open answer
Q14	Understanding Occupational Risks Through Data Visualisation	What are the main workplace risks that you are aware of in your daily activity?	Open answer
Q15	Understanding Occupational Risks Through Data Visualisation	Do you think you receive enough information about the occupational risks of your job?	Likert Scale: (1) Not at all (2) Slightly (3) Moderately (4) Very (5) Completely
Q16	Understanding Occupational Risks Through Data Visualisation	If you had access to occupational risk visualisations, what kind of information would be most useful?	Multiple choice: (1) Physical activity levels (2) Postural risk (3) Musculoskeletal risks (4) Environmental risks (e.g., ambient noise) (5) Other—option to specify
Q17	Understanding Occupational Risks Through Data Visualisation	How would you like information about workplace risks to be presented?	Multiple choice: (1) Personal risk reports (for personal use only) (2) Summaries by team/department (only average values to ensure employee privacy) (3) General trends in the organisation (4) Other—option to specify
Q18	Understanding Occupational Risks Through Data Visualisation	If a visualisation showed that your work habits increased the risks to your health, would you take any action? If so, what kind of action would you take?	Open answer
Q19	Collaboration between Stakeholders and Decision-Making	If you have received reports on occupational health in the workplace, how was the information presented?	Open answer
Q20	Collaboration between Stakeholders and Decision-Making	Have you ever had to explain health data to someone else? If so, what challenges did you face?	Open answer
Q21	Collaboration between Stakeholders and Decision-Making	How do you think health data visualisations can help improve communication between workers, health professionals and managers?	Open answer
Q22	Final Comments	If you have any comments, suggestions or any information that you think is relevant and could contribute to the development of occupational health data visualisations, this is the place to share it. You can write in any format you feel most comfortable with (e.g., long text, bullet points, etc.).	Open answer

### Appendix A.2. Co-Design Survey Questionnaire Results

The results are presented below, organised according to the five thematic sections of the questionnaire: (1) General Information, (2) Current Challenges and Needs, (3) Data Interpretation and Visualisation Preferences, (4) Understanding Occupational Risks Through Data Visualisation, and (5) Collaboration between Stakeholders and Decision-Making.

#### Appendix A.2.1. General Information

Respondents held the following roles within CML: customer service manager (n=5), ergonomist or occupational health specialist (n=2), clinician (n=1), clinical psychologist (n=1), and occupational safety technician (n=1). Participants reported a mean of 18.1±10.5 years of experience within CML (range: 1–30 years; median: 21 years), indicating a predominantly experienced cohort.

With respect to health data consultation habits (Q3), responses were distributed across multiple frequencies. The most common response was *every six months* (n=4), followed by *daily* and *once a week* (n=2 each), and *once a year* and *once a month* (n=1 each). Medical appointments (n=8) were the most consistently cited source of health information (Q4), followed by wearables (n=6) and health apps (n=5), while workplace reports were mentioned by three respondents. Two respondents stated using books and the internet as additional sources. When asked about the types of data monitored through health or fitness applications (Q5), physical activity levels (e.g., step count) and heart rate were the most frequently reported categories (n=5 and n=4, respectively), followed by sleep patterns (n=3) and stress indicators (n=2). Three participants reported not using any health or fitness applications.

#### Appendix A.2.2. Current Challenges and Needs

The majority of respondents reported finding it easy or very easy to interpret received health data (Q6). Specifically, six participants rated the task as *easy*, one as *very easy*, and three as *neutral*. No participant rated interpretation as difficult or very difficult. With regards to facing challenges when trying to understand health data (Q7), participants reported consistently not having any major challenges.

Despite reporting relatively high ease of interpretation, respondents identified several factors that they found confusing in health data visualisations (Q8). The most frequently cited issue was *lack of explanation* (n=4), followed by *confusing visual elements* and *difficulty relating the visuals to one’s own situation* (n=2 each), and *too much information* (n=1). One respondent noted that health information was fragmented across multiple systems and expressed a preference for centralising it within a single application.

When asked what they would change about the way health data is currently presented (Q9), respondents converged on three recurring themes. First, personalisation was emphasised, with multiple participants noting that data should be more closely related to the individual user’s condition and situation. Second, language and vocabulary were identified as barriers, with several respondents indicating that the terminology used in health data presentations should be simplified. Third, conciseness was noted as a desirable property, with participants requesting more succinct and actionable presentations.

#### Appendix A.2.3. Data Interpretation and Visualisation Preferences

The preferred types of data visualisation reported by respondents (Q10) were colour-coded risk levels, being the most preferred format (n=6), followed by charts such as bar charts and line graphs (n=5), simplified numerical values with explanation (n=4), and tables (n=2).

Among those who had previously used a health or fitness application (Q11; n=5 substantive responses), the most useful visualisations cited included heart rate oscillations and blood pressure trends, step count and physical activity summaries, sleep quality indicators, and simplified numerical values contextualised against reference thresholds.

All respondents who provided a substantive answer to Q12 (n=10) expressed a preference for *periodic summaries* over real-time updates. Among these, seven preferred weekly summaries and three preferred daily summaries. Reasons cited for this preference included the practical relevance of weekly overviews for detecting day-to-day variability across working and rest days, as well as the motivational value of daily feedback for sustaining behaviour change.

With regard to factors that would make occupational health visualisations easier to understand (Q13; n=3 substantive responses), respondents noted the importance of clarity, conciseness, and actionability. One participant emphasised that visualisations should explicitly demonstrate the influence of identified risk factors on the probability and severity of injury or disease development, while another expressed a preference for a single, unified application rather than data spread across multiple platforms.

#### Appendix A.2.4. Understanding Occupational Risks Through Data Visualisation

Respondents identified a range of occupational risks in their daily activities (Q14). The most frequently mentioned categories were psychosocial risks, including occupational stress, moral harassment, interpersonal conflicts, and mental health deterioration. Furthermore, postural and ergonomic risks associated with prolonged seated computer work, including repetitive movements, visual fatigue, and thermal discomfort, were mentioned. Musculoskeletal complaints, specifically tendinitis of the wrist and shoulder, and general postural problems, were also indicated as significant risks. Physical aggression was cited by one participant working in a front-office customer-facing role. Organisational factors such as excessive workload, poor information management, and inadequate management practices were also noted.

Responses to Q15, which assessed the perceived sufficiency of information received about occupational risks, spread across the full scale: two participants rated sufficiency as *not at all sufficient*, one as *slightly sufficient*, three as *neutral*, one as *sufficient*, and two as *very sufficient*. One respondent did not answer this question.

Musculoskeletal risks were considered most useful in occupational risk visualisations (Q16), with seven respondents selecting this option, followed by postural risk and environmental factors such as ambient noise (n=6 each), and physical activity levels (n=5). Psychosocial risks were suggested by two respondents as an additional option.

Regarding respondents’ preferences with respect to how occupational risk information should be presented (Q17), team or department-level summaries based on average values were the most frequently selected format (n=6), followed equally by personal risk reports for individual use and general organisational trends (n=5 each). These results suggest a preference for multi-level reporting, where individual, team-level, and organisational perspectives are presented in parallel.

When asked whether they would take action if a visualisation indicated that their work habits posed a health risk (Q18; n=8 substantive responses), all respondents indicated that they would act. The nature of the intended actions varied in specificity: responses ranged from general affirmations of intent to modify harmful habits, to more concrete expressions of willingness to change working conditions and posture, or to invest in eliminating identified risks. One participant acknowledged the difficulty of sustaining behavioural change, noting that, despite recognising existing risk factors, returning to habitual risk-associated behaviours was common.

#### Appendix A.2.5. Collaboration Between Stakeholders and Decision-Making

Among the four respondents who had previously received occupational health reports in the workplace (Q19), the formats cited included Word and Excel documents, formal written reports, the organisational intranet, and paper-based documents. No respondent reported having received interactive or digital visualisation-based reports, suggesting that current occupational health reporting practices within the studied organisation remain primarily document-centric.

Three respondents indicated prior experience explaining health data to others (Q20). Two of these identified challenges: one noted difficulties arising from the language and terminology used in health data, while the other highlighted the challenge of clarifying the correlations between data variables and their direct influence on health outcomes. One respondent reported no particular challenges.

Respondents who provided a substantive answer to Q21 (n=7 after excluding uninformative responses) generally expressed a positive view of the potential role of health data visualisations in improving communication. The most recurrently expressed themes were: (1) shared access to a common information base enabling alignment between workers, health professionals, and managers; (2) the communicative power of visual representations in clarifying data and facilitating shared understanding; and (3) the role of data confrontation, that is, presenting concrete numerical evidence of risk, as a driver of improvement proposals and intervention. One respondent cautioned that visualisations alone would be insufficient, emphasising the need for organisational structures to be oriented toward occupational health in order for any tool to have a meaningful impact.

A single respondent provided a comment in the final open-answer section (Q22), reiterating the importance of organisational commitment to occupational health and noting that the effectiveness of any health-oriented initiative, regardless of the engagement of individual managers, ultimately depends on the degree to which the broader organisational structure supports it.
